# Ivacaftor pharmacokinetics and lymphatic transport after enteral administration in rats

**DOI:** 10.3389/fphar.2024.1331637

**Published:** 2024-02-20

**Authors:** Jiří Pozniak, Pavel Ryšánek, David Smrčka, Petr Kozlík, Tomáš Křížek, Jaroslava Šmardová, Anežka Nováková, Debanjan Das, Daniel Bobek, Mahak Arora, Jiří Hofmann, Tereza Doušová, Martin Šíma, Ondřej Slanař

**Affiliations:** ^1^ Third Department of Surgery, First Faculty of Medicine, Motol University Hospital, Charles University, Prague, Czechia; ^2^ First Faculty of Medicine, Institute of Pharmacology, General University Hospital in Prague, Charles University, Prague, Czechia; ^3^ Zentiva, K. S, Prague, Czechia; ^4^ Department of Analytical Chemistry, Faculty of Science, Charles University, Prague, Czechia; ^5^ Department of Pediatrics, Second Faculty of Medicine, Motol University Hospital, Charles University, Prague, Czechia

**Keywords:** pharmacokintetics, lymphatic transport, ivacaftor, bioavailability, intestinal absorption

## Abstract

**Background:** Ivacaftor is a modern drug used in the treatment of cystic fibrosis. It is highly lipophilic and exhibits a strong positive food effect. These characteristics can be potentially connected to a pronounced lymphatic transport after oral administration.

**Methods:** A series of studies was conducted to describe the basic pharmacokinetic parameters of ivacaftor in jugular vein cannulated rats when dosed in two distinct formulations: an aqueous suspension and an oil solution. Additionally, an anesthetized mesenteric lymph duct cannulated rat model was studied to precisely assess the extent of lymphatic transport.

**Results:** Mean ± SD ivacaftor oral bioavailability was 18.4 ± 3.2% and 16.2 ± 7.8%, respectively, when administered as an aqueous suspension and an oil solution. The relative contribution of the lymphatic transport to the overall bioavailability was 5.91 ± 1.61% and 4.35 ± 1.84%, respectively.

**Conclusion:** Lymphatic transport plays only a minor role in the process of ivacaftor intestinal absorption, and other factors are, therefore, responsible for its pronounced positive food effect.

## Introduction

Ivacaftor is a medication used in the treatment of cystic fibrosis. It is a CFTR (cystic fibrosis transmembrane conductance regulator) protein potentiator that acts by increasing the open probability of the CFTR channel, thus enhancing the transport of chloride ions across epithelial membranes (Deeks, 2013). It was registered as the first drug of its class in 2012. Later, ivacaftor combinations with CFTR correctors lumacaftor and tezacaftor were registered (Deeks, 2016; Paterson et al., 2020). The latest combined pill that has been introduced to the market contains a combination of elexacaftor, ivacaftor, and tezacaftor as its active ingredients, where elexacaftor is a next-generation CFTR corrector (Hoy, 2019).

Ivacaftor, when used in monotherapy in adults, is administered orally at a dose of 150 mg twice daily (EMA, 2012). It is absorbed at a moderate speed with a T_max_ of 2–3 h. The Absolute oral bioavailability in humans is not known. Ivacaftor distributes extensively into the peripheral tissues with an apparent volume of distribution of 250–350 L. The drug is eliminated mainly in feces as metabolites formed via CYP3A. After several weeks of treatment, ivacaftor improves the respiratory function and health-related quality of life in patients with cystic fibrosis. It is generally well tolerated, with headache, oropharyngeal pain, upper respiratory tract infection, and nasal congestion being the most common adverse effects.

Ivacaftor is a highly lipophilic compound. Different computational algorithms (Chemaxon and ALOGPS) predict a log *p*-value between 5.0 and 5.8. It was shown that compounds with log *p* > 5 are typically absorbed into intestinal lymph to a large extent (Charman and Stella, 1986; Porter et al., 2007; Rysanek et al., 2020). Lymphatic transport after oral administration can have a major impact on the pharmacological properties of the drug. It can increase systemic bioavailability because the intestinal lymph vessels are an additional gate to the systemic blood circulation besides the standard blood vessel system of the portal vein. Furthermore, the intestinal lymph completely evades the first-pass metabolism in the liver because it directly enters the systemic blood. Finally, drugs that are targeted against the cells of the immune system (e.g., anti-inflammatory drugs, immunosuppressants, anti-HIV medicines, and some anti-cancer drugs ) can have an increased effect when absorbed through the intestinal lymphatic system, which contains all the typical cells and tissues like B and T lymphocytes, lymphatic follicles, and lymph nodes. While ivacaftor probably does not have a meaningful effect on the cells of the immune system, its lymphatic transport could play a role in maintaining systemic bioavailability by evading CYP3A4-mediated liver first-pass metabolism.

Bioavailability of ivacaftor is strongly enhanced by high-fat food. When administered with fat-containing food, the bioavailability increases by 2.5- to 4-fold (EMA, 2012). As a result, ivacaftor is recommended to be taken in fed state only. While the mechanisms behind the food effect are not known, such a pronounced effect of food on bioavailability is typically observed in drugs that are lymphatically transported, such as the anti-cancer agent venetoclax (Choo et al., 2014; Salem et al., 2016) and the calcimimetic drug cinacalcet (Padhi et al., 2007; Rysanek et al., 2021). The presence of food, especially when rich in fat, helps to solubilize the highly lipophilic drugs within the gastrointestinal tract (Koziolek et al., 2019), and additionally, the lipids play a crucial role as constituents of lipoproteins (chylomicrons), which serve as drug carriers in the intestinal lymph. The possible involvement of lymphatic transport may allow the development of lymph-targeting drug formulations to improve the bioavailability and PK performance.

There have already been successful attempts to increase the oral bioavailability of ivacaftor and reduce its positive food effect. In a well-designed, four-period, cross-over pharmacokinetic study in Beagle dogs, a self-nanoemulsifying drug delivery system (SNEDDS) formulation with a double-headed miscellaneous lipid increased the oral bioavailability by 7-fold compared to a simple suspension when administered in the fasted state (Miao et al., 2022). In the fed state, there was still a ∼50% increase in bioavailability, and most importantly, the bioavailability of ivacaftor after SNEDDS administration in the fasted and fed states was almost identical, i.e., the positive food effect was effectively eliminated. The underlying mechanisms were better active substance solubilization and improved drug release. The authors also hypothesized the possible contribution of increased lymphatic transport, which was, however, not studied. Lymphatic transport of ivacaftor has not been determined *in vivo* in any experimental species so far.

The aim of this study was to assess if the food-dependent bioavailability of ivacaftor is mediated via lymphatic transport. Therefore, ivacaftor pharmacokinetics with a special focus on lymphatic transport in rats was studied.

## Materials and methods

### Chemicals

Amorphous ivacaftor (Glenmark pharmaceuticals), methylcellulose 1500 mPa s, sodium dodecyl sulfate >99% (Carl Roth GmbH + Co. KG), polyethylene glycol (average M_n_ 600, Sigma-Aldrich), ethanol 96% for UV (Penta), castor oil Ph. Eur. grade (Sigma-Aldrich), and demineralized water.

Xylazine 20 mg mL^−1^ solution (Rometar^®^, Bioveta a. s., Czech Republic), ketamine 100 mg mL^−1^ solution (Narkamon^®^, Bioveta a. s., Czech Republic), and isoflurane (IsoFlo^®^, Zoetis/Pfizer, Czech Republic) were used for animal anesthesia. Ketoprofen 100 mg mL-1 (Ketodolor^®^, Le Vet Beheer B.V., the Netherlands) was used as an analgesic. Heparin solution 5,000 IU mL^−1^ (Zentiva k.s., Czech Republic) was used for catheter patency maintenance. T61^®^ (Intervet International B.V., the Netherlands) was used for animal euthanasia at the end of the experiments.

### Ivacaftor dosing forms

Previously, it was shown that the presence of lipids (especially long-chain triglycerides) in the dosing formulations stimulates the lymphatic transport of drugs (Caliph et al., 2000; Khoo et al., 2003). In order to fully elucidate ivacaftor’s potential for lymphatic transport, a lipid-containing formulation (oil solution) was prepared and compared to a lipid-free formulation (aqueous suspension).

Aqueous suspension: sodium dodecyl sulfate was dissolved in demineralized water to obtain a solution in which crude ivacaftor was dispersed. Methylcellulose was added while mixing the suspension to obtain a final vehicle with 0.5% (w/v) sodium dodecyl sulfate, 0.5% (w/v) methylcellulose, and 0.4% (w/v) ivacaftor.

Oil solution: ivacaftor was suspended in castor oil. The suspension was left overnight on laboratory shaker at 750 rpm and 37°C temperature until crude ivacaftor was fully dissolved. The concentration of dissolved ivacaftor was 0.4% (w/v).

Intravenous solution: PEG 400 was mixed with demineralized water and homogenized with pure ethanol. Ivacaftor was dissolved in the prepared vehicle to obtain a solution containing 20% (w/v) ethanol, 15% (w/v) PEG 600, and 0.25% (w/v) ivacaftor.

### Animals

All animal experiments were performed under approval from the Ministry of Education, Youth, and Sports, Czech Republic (MSMT-9445/2018–8). All efforts were made to minimize animal suffering. Male Wistar rats (weight 300–450 g and age 3–5 months) were purchased from Velaz s. r. o., Prague, Czech Republic. They were housed under the standard conditions (12-h light/dark cycle, 22°C temperature, and 50% humidity) in cages with wood shavings bedding (two rats per cage during acclimation and one rat per cage during experiment) and fed on water and granulated diet *ad libitum*. The acclimation period took at least 1 week. The animals were randomly assigned to the experimental groups.

### Pharmacokinetic studies

Ivacaftor’s absolute oral bioavailability and other pharmacokinetic parameters for aqueous suspension and oil solution were determined in two two-period, one-sequence (IV-PO), cross-over studies. The cross-over study design was chosen because it decreases the impact of inter-individual variability and reduces the number of animals needed for each experiment (Kralovicova et al., 2022). It was already successfully implemented in numerous studies in the recent years (Boleslavska et al., 2020; Hrinova et al., 2022; Jelinek et al., 2022; Salamunova et al., 2023).

The animals were anaesthetized with i.m. xylazine (5 mg kg^−1^) and ketamine (100 mg kg^−1^) after a rapid isoflurane induction. Both the jugular veins were cannulated (3 Fr polyurethane catheter, Instech Laboratories, Plymouth Meeting, United States). One catheter was used for i.v. dosing and the second one for repetitive blood sampling in order to avoid sampling cannula contamination with highly concentrated intravenous drug solution. After a 3-day recovery period, the rats were i.v. dosed with ivacaftor 0.25 mg (volume 100 µL). Systemic blood (100 µL per sample) was drawn at 5, 15, and 30 min and at 1, 2, 4, 6, 10, and 24 h(s) post-dose. After a 3-day wash-out period, the oral dosing followed. Ivacaftor 4 mg (volume 1 mL) was administered in the form of an aqueous suspension or an oil solution. Systemic blood (100 µL per sample) was drawn at 0 (pre-dose), 1, 2, 3, 4, 5, 6, 8, 10, 24, and 30 h(s).

### Lymphatic transport studies

A mesenteric lymph duct cannulated and anaesthetized rat model was used as previously described with slight modifications (Trevaskis et al., 2015). Rats were left on normal diet and given 0.5 mL olive oil 1 hour prior to surgery to visualize the mesenteric lymph (milky white color). They were anaesthetized with i.m. xylazine (5 mg kg^−1^) and ketamine (100 mg kg^−1^) after a rapid isoflurane induction. Transverse laparotomy was performed. Mesenteric duct was identified cranially to superior mesenteric artery and cannulated with heparin prefilled 0.97 mm outer diameter and 0.58 mm inner diameter polyethylene catheter (Instech Laboratories, Plymouth Meeting, United States). The catheter was fixed in place with two to three drops of tissue adhesive (Histoacryl^®^, B. Braun Surgical, S.A., Spain). A duodenal catheter was also placed (same parameters as for lymphatic catheter) via a small duodenotomy and fixed with tissue adhesive. The abdominal wall was sutured in two layers, with both catheters leaving the abdominal cavity through the right flank. At the end of the procedure, the right jugular vein was cannulated for blood sampling.

The rats were then placed on heated pads and covered with a blanket to prevent heat loss. Ivacaftor (4 mg in the form of an aqueous suspension or an oil solution) was dosed slowly via the duodenal catheter over 30 min. The administered volume was 1 mL. Whole lymph was collected in regularly changed Eppendorf tubes from the time the dosing started. When the dosing was finished, continuous hydration with normal saline at a rate of 3 mL h^−1^ intraduodenally followed using an infusion pump (Perfusor^®^ compactplus, B. Braun Melsungen AG, Germany). Anesthesia was maintained throughout the rest of the experiment, and additional ketamine i. m. boluses were given whenever necessary. Eppendorf tubes were changed hourly, and systemic blood was drawn at the same time points until 8 hours post-dose.

### Sample processing

Blood samples were centrifuged (4,500 rpm for 10 min), and serum was extracted. Lymph volume was measured gravimetrically, and the samples were further processed without additional adjustment. All samples were stored in -80°C until analysis. The laboratory was unaware of animal assignment to particular experimental group (laboratory blinding).

### Analytical methods

Ivacaftor concentration was analyzed in both the serum and lymph samples. Protein precipitation was employed for extracting the serum and lymph samples. The procedure involved adding 60 µL of 100% acetonitrile, containing 50.0 ng/mL of Ivacaftor-d19 as an internal standard, to 15 µL of the sample. The mixture underwent vortexing and centrifugation at 10,000 *g* for 10 min. Subsequently, 50 µL of the supernatant was transferred into a vial for liquid chromatography–mass spectrometry (LC–MS) analysis. For the LC–MS analysis, a Shimadzu UHPLC Nexera X3 system connected to a Triple Quad 8045 tandem mass spectrometer was utilized. Chromatographic separation was conducted on an Acquity UPLC BEH C18 column (50 × 2.1 mm; 1.7 µm) from Waters. The mobile phase consisted of 0.1% formic acid in deionized water (solvent A) and methanol with 0.1% formic acid (solvent B). The flow rate was maintained at 0.45 mL/min, with an injection volume of 1 µL. Column temperature was set at 40°C, and the samples were maintained at 10°C. The optimized gradient elution followed this pattern (time/% B): 0/50, 0.5/50, 2.5/90, 3.5/90, 4.0/50, and 6.0/50. The mass spectrometer was operated in the positive mode with multiple reaction-monitoring (MRM) settings. Electrospray ion source conditions included nebulizing gas flow of 3 L/min, heating gas flow of 10 L/min, interface temperature at 325°C, desolvation line temperature at 225°C, heat-block temperature at 400°C, and drying gas flow at 8 L/min. MRM transitions of 393.0 > 172.0 (Q1 pre-bias -20 V, Q3 pre-bias -32 V, and collision energy -28 V) and 412.2 > 172.0 (Q1 pre-bias -20 V, Q3 pre-bias -30 V, and collision energy -30 V) were monitored for ivacaftor and ivacaftor-d19, respectively.

Calibration curves were generated separately for each matrix (serum and lymph). These curves encompassed seven concentrations and were constructed by plotting the ratio of the ivacaftor peak area to the internal standard’s peak area against ivacaftor concentration. The weighted least-squares linear regression method was employed using a weighting factor of 1/x^2 to enhance the accuracy at low concentrations. LLOQ, which was the lowest calibration standard, was 10 ng mL^−1^ with a precision and accuracy of up to 12% (back-calculated). LLOQ MRM chromatograms are depicted in Supplementary Figure S1. ULOQ, which was the highest calibration standard, was 2,000 ng mL^−1^ with a precision and accuracy of up to 5% (back-calculated). The accuracy and precision of back-calculated concentrations of other calibration points were within 4% of the nominal concentration. The method demonstrated linearity (coefficients of determination, *R* ^2^, exceeding 0.9998) within the concentration range of 10–2,000 ng/mL. Method accuracy and precision were evaluated by measuring five replicates at four different concentrations of QC samples (10, 30, 800, and 2,000 ng mL^−1^) on two different days in each matrix. The accuracy and precision results are presented in Supplementary Table S1. The inter-day and intra-day precisions (RSD %) ranged from 2.5% to 8.5%, and the accuracy (RE %) was within ±11.5% for the serum matrix. For the lymph matrix, the inter-day and intra-day precision was up to 8.1%, and accuracy was within ±11.2%. The recovery of the method was checked by the comparison of the area of the ivacaftor peak (as well as IS) in a matrix sample spiked with the standard before the precipitation of proteins, and the area in a matrix sample spiked after the precipitation of proteins at three concentration levels (10, 800, and 2,000 ng mL^−1^). The recovery for ivacaftor and IS ranged from 97.2% to 101.5% for both matrices. The developed method is selective because MRM chromatograms of the blank matrices showed no interfering compound within the retention time window of ivacaftor and IS (Supplementary Figure S1). Validation of the method was carried out according to the criteria stipulated in the European Medicines Agency (EMA) guidelines on bioanalytical method validation (EMA, 2022). All validation parameters met the acceptance criteria outlined in the guideline.

### Data analysis and statistics

Serum and lymph concentrations in all studies were dose normalized to 1 mg kg^−1^ prior to further calculations. The AUC values were determined using the linear trapezoidal rule. Exact actual sampling times were used for this purpose. Scheduled sampling times were used for mean concentration plotting in the graphs. PK-solver add-on for MS Excel was used for all basic pharmacokinetic calculations (Zhang et al., 2010). GraphPad Prism version 10.0.2 (GraphPad Software, San Diego, CA, United States) was used for all statistical analyses and graph plotting. Unpaired Student’s t-test was used to compare the pharmacokinetic and lymphatic transport parameters between the experimental groups. Level of significance was set at *p* < 0.05.

### Calculation of lymphatic transport parameters

Lymphatic transport parameters were defined and calculated as previously described (Rysanek et al., 2020; Rysanek et al., 2021; Jelinek et al., 2022; Salamunova et al., 2023). Briefly, absolute bioavailability via lymph (F_**AL**_ **)** was defined as the percentage of the administered drug dose absorbed into the lymph. It was determined directly from the lymph volume and drug concentration in lymph duct cannulated rats. Absolute bioavailability via portal vein (F_AP_) was analogically defined as the percentage of the administered drug dose reaching the systemic circulation after direct absorption into blood. It was calculated using the following equation:

$$F_{\text{AP}}={\text{AUC}}_{\text{ent}}\times {\text{AUC}}_{\text{iv}}^{-1},$$ (1)

where AUC_ent_ is the area under the dose-normalized blood concentration–time curve after enteral dosing in lymph duct cannulated (i.e. lymph deprived) rats and AUC_iv_ is the respective parameter in a separate intravenously dosed group. Total absolute bioavailability (F) in lymph duct cannulated rats was calculated as a sum of F_AL_ and F_AP_. In normal animals with no lymph duct cannulation, F was calculated using the standard formula for oral bioavailability:

$$F={\text{AUC}}_{\text{po}}\times {\text{AUC}}_{\text{iv}}^{-1}.$$ (2)

Relative bioavailability via lymph (F_RL_) was defined as the percentage of the systemically available drug that was absorbed via lymph. It was calculated using the following equation:

$$F_{\text{RL}}=F_{\text{AL}}\times F^{-1}.$$ (3)

## Results

### Pharmacokinetic studies

**FIGURE 1:**
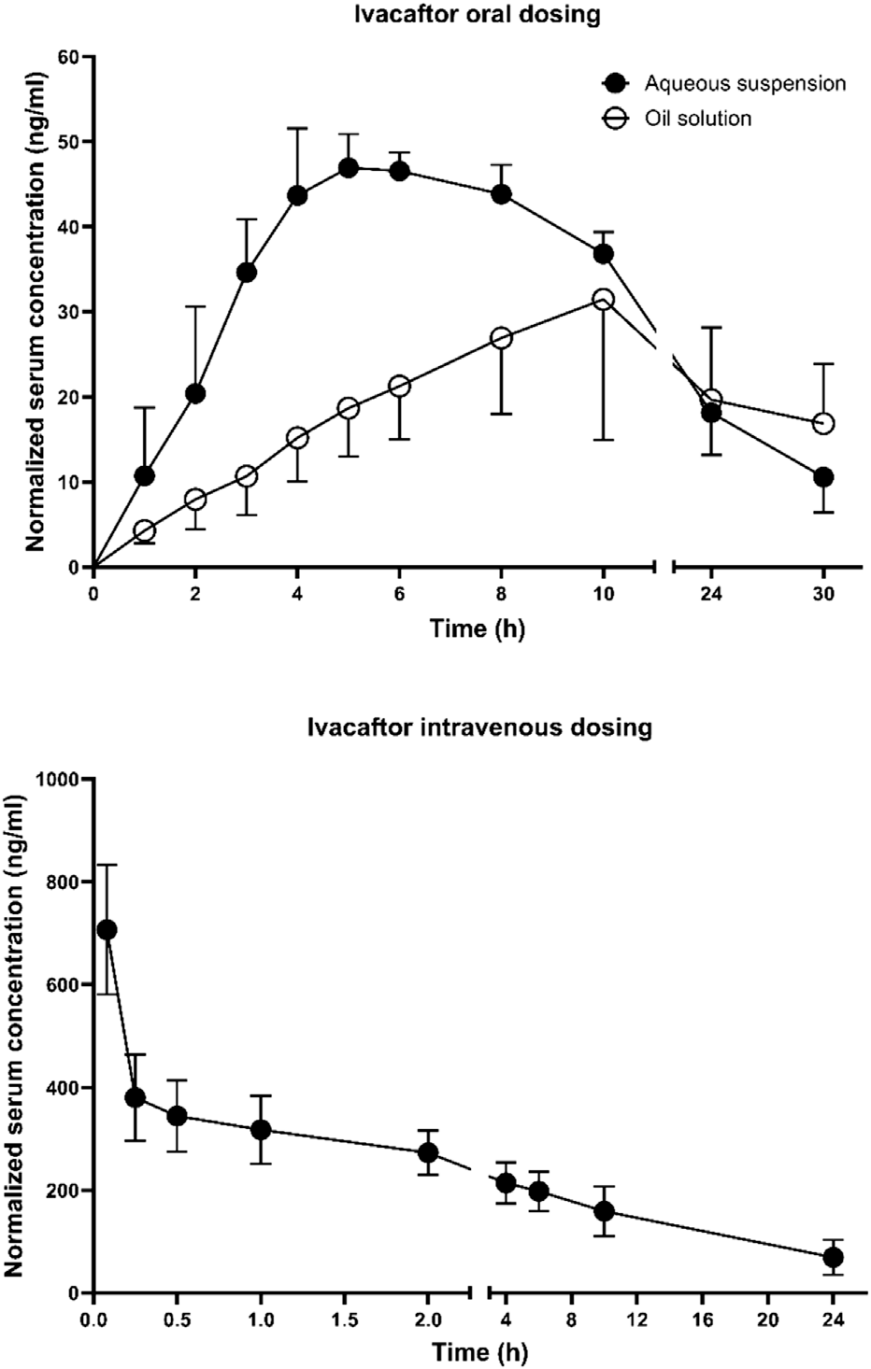
Mean ± SD serum pharmacokinetic profiles after oral administration of ivacaftor 4 mg in the form of an aqueous suspension (n = 5) and an oil solution (n = 7) and intravenous administration of ivacaftor 0.25 mg (n = 13). The profiles are derived from two two-period (IV-PO) cross-over studies. All concentrations are dose-normalized to 1 mg kg^−1^.

Ivacaftor’s pharmacokinetic profiles after oral and intravenous administration are shown in Figure 1. Table 1 contains all the basic pharmacokinetic parameters. Ivacaftor did absorb slowly from both the formulations, with T_max_ of 5 h for the aqueous suspension and ≥ 8 h for the oil solution. T_max_ for the oil solution could not be determined accurately due to missing sampling times after 10 h. The ivacaftor oral bioavailability was ∼20% and did not differ significantly between the two formulations. The elimination was slow, with a clearance of 0.2 L h^−1^ kg^−1^ and half-life of 12 h after intravenous dosing.

**TABLE 1:** Mean ± SD serum pharmacokinetic parameters after oral administration of ivacaftor 4 mg in the form of an aqueous suspension and an oil solution and intravenous administration of ivacaftor 0.25 mg. C_max_ and AUC_0-inf_ are dose-normalized to 1 mg kg^−1^. T_1/2_, AUC_0-inf_, and F_0-inf_ in the oil solution group could not be assessed due to late occurrence of T_max_, *p < 0.05 vs. aqueous suspension, ***p < 0.001 vs. aqueous suspension.

	Aqueous suspension (n = 5)	Oil solution (n = 7)	Intravenous dosing (n = 13)
C_max_ (ng mL^−1^)	50 ± 4	33 ± 14*	707 ± 121
T_max_ (h)	5.06 ± 0.86	8.85 ± 1.45***	-
T_1/2_ (h)	12.1 ± 3.7	-	12.2 ± 4.7
AUC_0-24_ (ng h mL^−1^)	734 ± 45	532 ± 197	3,923 ± 893
AUC_0-inf_ (ng h mL^−1^)	1,023 ± 192	-	5,357 ± 2,207
F_0-24_ (%)	18.4 ± 3.2	16.2 ± 7.8	-
F_0-inf_ (%)	19.7 ± 3.9	-	-
V_ss_ (L kg^−1^)	-	-	3.13 ± 0.53
CL (L h^−1^ kg^−1^)	-	-	0.21 ± 0.07

### Lymphatic transport studies

**FIGURE 2:**
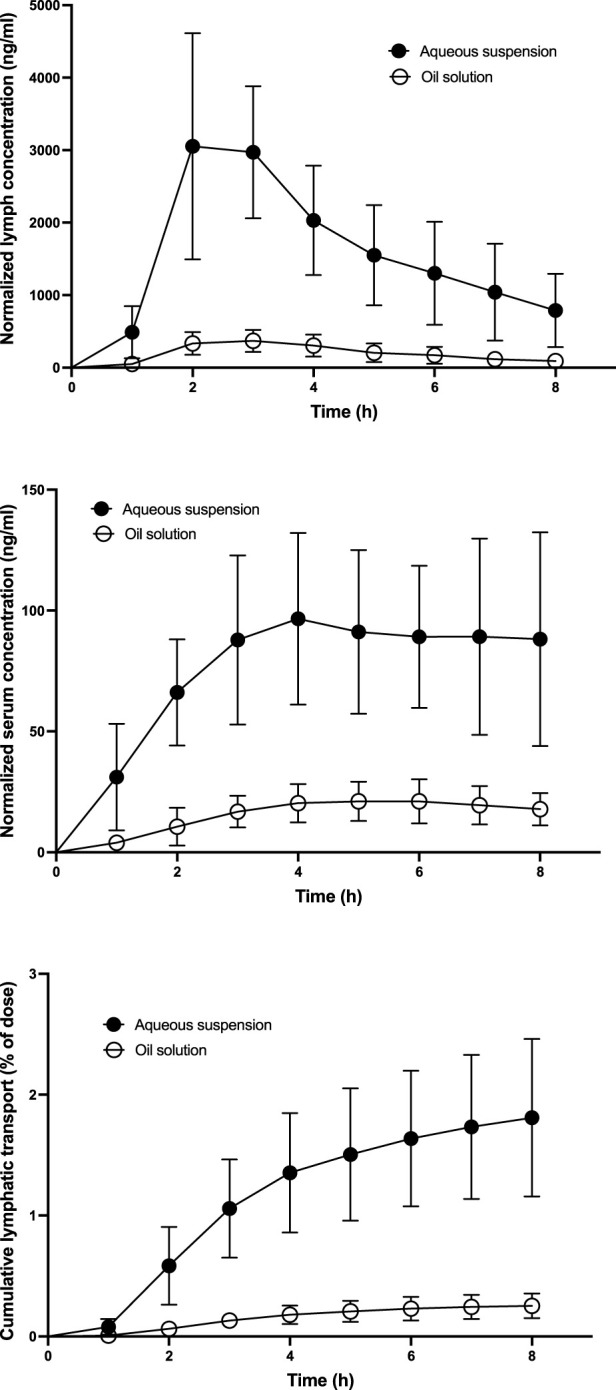
Mean ± SD ivacaftor lymph and serum pharmacokinetic profiles and cumulative lymphatic transport assessed after administration of ivacaftor 4 mg in the form of an aqueous suspension and an oil solution (n = 6 for both) to lymph duct cannulated rats. All concentrations are dose-normalized to 1 mg kg^−1^.

Ivacaftor serum and lymph pharmacokinetic profiles and cumulative lymphatic transport measured in the lymph duct cannulated rats are shown in Figure 2. Table 2 contains the calculated lymphatic transport parameters. Ivacaftor bioavailability and bioavailability via lymph was significantly higher after the administration of the aqueous suspension compared to the oil solution. Nevertheless, the relative bioavailability via lymph (F_RL_) of ∼5% was similar in both formulations. Hence, the relative contribution of lymphatic transport to the overall ivacaftor bioavailability was rather low.

**TABLE 2:** Mean ± SD ivacaftor lymphatic transport parameters after intraduodenal administration of an aqueous suspension or an oil solution to lymph duct cannulated rats. F, bioavailability; F_AL_, absolute bioavailability via lymph; F_RL_, relative bioavailability via lymph; ***p < 0.001 vs. aqueous suspension.

	Aqueous suspension (n = 6)	Oil solution (n = 6)
AUC_0-8_ lymph (ng h mL^−1^)	12,832 ± 4,884	1,590 ± 657***
AUC_0-8_ blood (ng h mL^−1^)	596 ± 183	122 ± 46***
Lymph/blood AUC ratio	23.2 ± 9.6	14.4 ± 6.7
F (%)	31.8 ± 9.6	6.4 ± 2.3***
F_AL_ (%)	1.81 ± 0.59	0.25 ± 0.09***
F_RL_ (%)	5.91 ± 1.61	4.35 ± 1.84

## Discussion

The slow ivacaftor absorption with T_max_ ≥ 5 h and quite a long elimination half-life (12 h) correspond to the results published earlier in preclinical studies conducted in the course of the ivacaftor original drug product development (FDA, 2012). On the other hand, the oral bioavailability of ∼20% in rats is lower than expected (40%–65%) despite the administration of a very similar formulation (0.5% methylcellulose suspension). The reason for this disproportion could be the cross-over study design implemented in this study as opposed to the classical parallel design, where indirect comparison with the data from only one intravenously dosed group are used to calculate bioavailability for all orally dosed groups, and inter-subject variability may alter the results substantially. The ivacaftor absorption from the oil solution was very slow with T_max_ ≥ 8 h. The reason probably lies in slow solubilization and digestion of castor oil, which was used in the formulation. Castor oil hydrolyzes in the gastrointestinal tract producing ricinoleic acid, which does not absorb very well when present in large amounts (Watson and Gordon, 1962).

The ivacaftor lymphatic transport played only a minor role in the perspective of its general pharmacokinetics. The relative bioavailability via lymph (F_RL_) of ∼5% for both the formulations is comparable to some other highly lipophilic compounds with ‘borderline’ lymphatic transport, such as abiraterone (Rysanek et al., 2021), seocalcitol (Grove et al., 2006), and ontazolast (Hauss et al., 1998). The reason why the lymphatic transport was not significant (F_RL_ > 10%) may lie in borderline ivacaftor lipophilicity, with the predicted log P of around 5.0.

An interesting aspect of the lymphatic transport studies was the very low ivacaftor total bioavailability and absolute bioavailability via lymph (F_AL_) after the administration of the oil solution. The total bioavailability was several times lower than that observed in the standard pharmacokinetic study (6% vs. 16%). This difference can be explained by the anesthesia that was applied throughout the whole lymphatic transport study. Previously, it was shown that general anesthesia (and surgery) decreases the oral bioavailability of drugs (Dahan et al., 2007; Valtola et al., 2007). The underlying mechanism is presumably an inhibited function of the gastrointestinal tract, particularly the decreased motility and reduced production of digestive fluids. These factors affected the ivacaftor absorption from the oil solution more than from the aqueous suspension since it depends on solubilization and digestion of fats. Nevertheless, the anesthesia does not affect the relative bioavailability via lymph (F_RL_) (Dahan et al., 2007). Therefore, the anesthetized mesenteric lymph duct cannulated rat model is suitable for the lymphotropic behavior testing of drugs, and the values of ∼5% observed in this particular case of ivacaftor are valid.

As shown in this study, the lymphatic transport of ivacaftor is low even when administered as an oil solution. Therefore, other factors are responsible for its pronounced food effect in humans. In addition, lymphatic targeting is not a feasible strategy for ivacaftor oral bioavailability improvement and reduction of its substantial food dependency.

## Conclusions

Ivacaftor exhibits slow absorption from gastrointestinal tract and slow elimination. The oral bioavailability is moderate. Despite a pronounced lipophilicity, ivacaftor lymphatic transport plays only a minor role in the process of intestinal absorption and does not explain the large effect of food on the drug bioavailability.

## Data Availability

The raw data supporting the conclusion of this article will be made available by the authors, without undue reservation.

## References

[B1] Boleslavska T. Svetlik S. Zvatora P. Bosak J. Dammer O. Beranek J. (2020). Preclinical evaluation of new formulation concepts for abiraterone acetate bioavailability enhancement based on the inhibition of pH-induced precipitation. Eur. J. Pharm. Biopharm. 151, 81–90. 10.1016/j.ejpb.2020.04.005 32298757

[B2] Caliph S. M. Charman W. N. Porter C. J. (2000). Effect of short-medium-and long-chain fatty acid-based vehicles on the absolute oral bioavailability and intestinal lymphatic transport of halofantrine and assessment of mass balance in lymph-cannulated and non-cannulated rats. J. Pharm. Sci. 89 (8), 1073–1084. 10.1002/1520-6017(200008)89:8<1073::aid-jps12>3.0.co;2-v 10906731

[B3] Charman W. N. A. Stella V. J. (1986). Estimating the maximal potential for intestinal lymphatic transport of lipophilic drug molecules. Int. J. Pharm. 34 (1-2), 175–178. 10.1016/0378-5173(86)90027-x

[B4] Choo E. F. Boggs J. Zhu C. Q. Lubach J. W. Catron N. D. Jenkins G. (2014). The role of lymphatic transport on the systemic bioavailability of the bcl-2 protein family inhibitors navitoclax (ABT-263) and ABT-199. Drug Metab. Dispos. 42 (2), 207–212. 10.1124/dmd.113.055053 24212376

[B5] Dahan A. Mendelman A. Amsili S. Ezov N. Hoffman A. (2007). The effect of general anesthesia on the intestinal lymphatic transport of lipophilic drugs: comparison between anesthetized and freely moving conscious rat models. Eur. J. Pharm. Sci. 32 (4-5), 367–374. 10.1016/j.ejps.2007.09.005 17980560

[B6] Deeks E. D. (2013). Ivacaftor: a review of its use in patients with cystic fibrosis. Drugs 73 (14), 1595–1604. 10.1007/s40265-013-0115-2 24030637

[B7] Deeks E. D. (2016). Lumacaftor/ivacaftor: a review in cystic fibrosis. Drugs 76 (12), 1191–1201. 10.1007/s40265-016-0611-2 27394157

[B8] EMA (2012). Kalydeco - summary of product characteristics.

[B9] EMA (2022). ICH guideline M10 on bioanalytical method validation and study sample analysis.

[B10] FDA (2012). Addendum #2 - Pharmacology and toxicology secondary review for NDA 203188.

[B11] Grove M. Nielsen J. L. Pedersen G. P. Mullertz A. (2006). Bioavailability of seocalcitol IV: evaluation of lymphatic transport in conscious rats. Pharm. Res-Dordr 23 (11), 2681–2688. 10.1007/s11095-006-9109-z 17048118

[B12] Hauss D. J. Fogal S. E. Ficorilli J. V. Price C. A. Roy T. Jayaraj A. A. (1998). Lipid-based delivery systems for improving the bioavailability and lymphatic transport of a poorly water-soluble LTB4 inhibitor. J. Pharm. Sci. 87 (2), 164–169. 10.1021/js970300n 9519148

[B13] Hoy S. M. (2019). Elexacaftor/ivacaftor/tezacaftor: first approval. Drugs 79 (18), 2001–2007. 10.1007/s40265-019-01233-7 31784874

[B14] Hrinova E. Skorepova E. Cerna I. Kralovicova J. Kozlik P. Krizek T. (2022). Explaining dissolution properties of rivaroxaban cocrystals. Int. J. Pharm. 622, 121854. 10.1016/j.ijpharm.2022.121854 35623488

[B15] Jelinek P. Rousarova J. Rysanek P. Jezkova M. Havlujova T. Pozniak J. (2022). Application of oil-in-water cannabidiol emulsion for the treatment of rheumatoid arthritis. Cannabis Cannabinoid 2022, 176. 10.1089/can.2022.0176 PMC10874822 36342775

[B16] Khoo S. M. Shackleford D. M. Porter C. J. Edwards G. A. Charman W. N. (2003). Intestinal lymphatic transport of halofantrine occurs after oral administration of a unit-dose lipid-based formulation to fasted dogs. Pharm. Res. 20 (9), 1460–1465. 10.1023/a:1025718513246 14567642

[B17] Koziolek M. Alcaro S. Augustijns P. Basit A. W. Grimm M. Hens B. (2019). The mechanisms of pharmacokinetic food-drug interactions - a perspective from the UNGAP group. Eur. J. Pharm. Sci. 134, 31–59. 10.1016/j.ejps.2019.04.003 30974173

[B18] Kralovicova J. Bartunek A. Hofmann J. Krizek T. Kozlik P. Rousarova J. (2022). Pharmacokinetic variability in pre-clinical studies: sample study with abiraterone in rats and implications for short-term comparative pharmacokinetic study designs. Pharmaceutics 14 (3), 643. 10.3390/pharmaceutics14030643 35336017 PMC8955109

[B19] Miao Y. F. Zhao S. H. Zuo J. Sun J. Q. Wang J. N. (2022). Reduced the food effect and enhanced the oral bioavailability of ivacaftor by self-nanoemulsifying drug delivery system (SNEDDS) using a new oil phase. Drug Des. Dev. Ther. 16, 1531–1546. 10.2147/DDDT.S356967 PMC9143795 35637746

[B20] Padhi D. Salfi M. Harris R. Z. (2007). The pharmacokinetics of cinacalcet are unaffected following consumption of high- and low-fat meals. Am. J. Ther. 14 (3), 235–240. 10.1097/01.mjt.0000212703.71625.26 17515696

[B21] Paterson S. L. Barry P. J. Horsley A. R. (2020). Tezacaftor and ivacaftor for the treatment of cystic fibrosis. Expert Rev. Resp. Med. 14 (1), 15–30. 10.1080/17476348.2020.1682998 31626570

[B22] Porter C. J. H. Trevaskis N. L. Charman W. N. (2007). Lipids and lipid-based formulations: optimizing the oral delivery of lipophilic drugs. Nat. Rev. Drug Discov. 6 (3), 231–248. 10.1038/nrd2197 17330072

[B23] Rysanek P. Grus T. Lukac P. Kozlik P. Krizek T. Pozniak J. (2021). Validity of cycloheximide chylomicron flow blocking method for the evaluation of lymphatic transport of drugs. Brit J. Pharmacol. 178 (23), 4663–4674. 10.1111/bph.15644 34365639

[B24] Rysanek P. Grus T. Sima M. Slanar O. (2020). Lymphatic transport of drugs after intestinal absorption: impact of drug formulation and physicochemical properties. Pharm. Res. 37 (9), 166. 10.1007/s11095-020-02858-0 32770268

[B25] Salamunova P. Krejci T. Rysanek P. Salon I. Kroupova J. Hubatova-Vackova A. (2023). Serum and lymph pharmacokinetics of nilotinib delivered by yeast glucan particles *per os* . Int. J. Pharm. 634, 122627. 10.1016/j.ijpharm.2023.122627 36693484

[B26] Salem A. H. Agarwal S. K. Dunbar M. Nuthalapati S. Chien D. Freise K. J. (2016). Effect of low- and high-fat meals on the pharmacokinetics of venetoclax, a selective first-in-class BCL-2 inhibitor. J. Clin. Pharmacol. 56 (11), 1355–1361. 10.1002/jcph.741 27029823

[B27] Trevaskis N. L. Hu L. Caliph S. M. Han S. Porter C. J. (2015). The mesenteric lymph duct cannulated rat model: application to the assessment of intestinal lymphatic drug transport. J. Vis. Exp. 97, 52389. 10.3791/52389 PMC4401200 25866901

[B28] Valtola A. Kokki H. Gergov M. Ojanpera I. Ranta V. P. Hakala T. (2007). Does coronary artery bypass surgery affect metoprolol bioavailability. Eur. J. Clin. Pharmacol. 63 (5), 471–478. 10.1007/s00228-007-0276-6 17333158

[B29] Watson W. C. Gordon R. S. Jr (1962). Studies on the digestion, absorption and metabolism of castor oil. Biochem. Pharmacol. 11, 229–236. 10.1016/0006-2952(62)90078-3 14005307

[B30] Zhang Y. Huo M. R. Zhou J. P. Xie S. F. (2010). PKSolver: an add-in program for pharmacokinetic and pharmacodynamic data analysis in Microsoft Excel. Comput. Meth Prog. Bio 99 (3), 306–314. 10.1016/j.cmpb.2010.01.007 20176408

