# Supplementary material for: Ivacaftor pharmacokinetics and lymphatic transport after enteral administration in rats
Source: Front Pharmacol. 2024 Feb 20;15:1331637. doi: 10.3389/fphar.2024.1331637 (PMC10912587; doi:10.3389/fphar.2024.1331637)
Supplement: Supplementary file 1 [file Table1.DOCX]

**Fig. S1** Representative MRM chromatograms of Ivacaftor and associated IS obtained under optimized LC-MS/MS conditions. (A) A blank serum sample; (B) A blank serum sample spiked with Ivacaftor at the LLOQ concentration of 10 ng mL^-1^; (C) A blank lymph sample; (D) A blank lymph sample spiked with Ivacaftor at the LLOQ concentration of 10 ng mL^-1^.

**Tab. S1** Accuracy and precision of the LC-MS/MS method (*n* = 5 replicates; for 2 days)

| Ivacaftor concentration  (ng mL^-1^) | Intra-day (*n=5*) |  |  | Inter-day (*n=5*) |  |  |
| --- | --- | --- | --- | --- | --- | --- |
|  | Measured concentrations  (ng mL^-1^) (mean±SD) | RSD (%) | RE (%) | Measured concentrations  (ng mL^-1^) (mean±SD) | RSD (%) | RE (%) |
| Serum |  |  |  |  |  |  |
| 10 | 11.0 ± 0.9 | 7.8 | 10.3 | 11.2 ± 0.9 | 8.5 | 11.5 |
| 30 | 28.7 ± 1.8 | 4.1 | -4.1 | 31.4 ± 1.5 | 4.8 | 4.5 |
| 800 | 824.8 ± 20.6 | 2.5 | 3.1 | 831.2 ± 24.1 | 2.9 | 3.9 |
| 2000 | 1930.0 ± 55.9 | 2.9 | -3.5 | 1942.0 ± 66.0 | 3.4 | -2.9 |
| Lymph |  |  |  |  |  |  |
| 10 | 10.9 ± 0.8 | 7.5 | 9.9 | 11.1 ± 0.9 | 8.1 | 11.2 |
| 30 | 28.6 ± 1.1 | 3.9 | -4.8 | 31.4 ± 1.2 | 3.9 | 4.7 |
| 800 | 830.4 ± 29.9 | 3.6 | 3.8 | 827.2 ± 25.6 | 3.1 | 3.4 |
| 2000 | 1902.0 ± 81.8 | 4.3 | -4.9 | 1918.0 ± 69.0 | 3.6 | -4.1 |

SD, standard deviation; RSD, relative standard deviation; RE, relative error (calculated as ((measured concentration - expected concentration)/expected concentration) × 100)
